# Rurality, Cardiovascular Risk Factors, and Early Cardiovascular Disease among Childhood, Adolescent, and Young Adult Cancer Survivors

**DOI:** 10.21203/rs.3.rs-4139837/v1

**Published:** 2024-04-01

**Authors:** David H. Noyd, Anna Bailey, Amanda Janitz, Talayeh Razzaghi, Sharon Bouvette, William Beasley, Ashley Baker, Sixia Chen, David Bard

**Affiliations:** Seattle Children’s Hospital/University of Washington Department of Pediatrics; The University of Oklahoma Health Sciences Center, Hudson College of Public Health, Department of Biostatistics and Epidemiology; The University of Oklahoma Health Sciences Center, Hudson College of Public Health, Department of Biostatistics and Epidemiology; The University of Oklahoma, School of Industrial and Systems Engineering; The University of Oklahoma Health Sciences Center, College of Medicine; The University of Oklahoma Health Sciences Center, College of Medicine; The University of Oklahoma Health Sciences Center, College of Medicine; The University of Oklahoma Health Sciences Center, Hudson College of Public Health, Department of Biostatistics and Epidemiology; The University of Oklahoma Health Sciences Center, College of Medicine

**Keywords:** Cardio-Oncology, Late Effects from Childhood Cancer, Survivorship, Clinical Research Informatics

## Abstract

**Background and Aims:**

Cardiovascular risk factors (CVRFs) later in life potentiate risk for late cardiovascular disease (CVD) from cardiotoxic treatment among survivors. This study evaluated the association of baseline CVRFs and CVD in the early survivorship period.

**Methods:**

This analysis included patients ages 0–29 at initial diagnosis and reported in the institutional cancer registry between 2010 and 2017 (n = 1228). Patients who died within five years (n = 168), those not seen in the oncology clinic (n = 312), and those with CVD within one year of diagnosis (n = 17) were excluded. CVRFs (hypertension, diabetes, dyslipidemia, and obesity) within one year of initial diagnosis, were constructed and extracted from the electronic health record based on discrete observations, ICD9/10 codes, and RxNorm codes for antihypertensives.

**Results:**

Among survivors (n = 731), 10 incident cases (1.4%) of CVD were observed between one year and five years after the initial diagnosis. Public health insurance (p = 0.04) and late effects risk strata (p = 0.01) were positively associated with CVD. Among survivors with public insurance(n = 495), two additional cases of CVD were identified from claims data with an incidence of 2.4%. Survivors from rural areas had a 4.1 times greater risk of CVD compared with survivors from urban areas (95% CI: 1.1–15.3), despite adjustment for late effects risk strata.

**Conclusions:**

Clinically computable phenotypes for CVRFs among survivors through informatics methods were feasible. Although CVRFs were not associated with CVD in the early survivorship period, survivors from rural areas were more likely to develop CVD.

**Implications for Survivors:**

Survivors from non-urban areas and those with public insurance may be particularly vulnerable to CVD.

## INTRODUCTION

Remarkable progress in the overall survival of children, adolescents, and young adults with cancer comes at a significant cost with late therapy-associated toxicities and there is a critical need for risk-based care. Seventy-four percent of survivors developed at least one chronic health condition in adulthood with a thirty-year cumulative incidence of 42% for a severe or life-threatening condition or death.^1^ Adolescents and young adults also face unique challenges after cancer treatment.^2,3^ Survivorship-focused, evidence-based care is essential to mitigate sequelae, such as heart failure and other cardiovascular diseases (CVD), to optimize the health of survivors and promote health equity.^4^

Cardiovascular risk factors (CVRFs) potentiate cardiotoxicity from anthracyclines and chest radiation among survivors. CVRFs later in life elevate the risk for CVD in a near multiplicative fashion, with an excess relative risk for heart failure of 44.5 due to the interaction of hypertension and anthracyclines.^5^ The inclusion of CVRFs, when added to treatment-related exposures, further refines risk prediction of subsequent heart failure among survivors.^6^ For adults with cancer, baseline cardiovascular risk assessment prior to initiation of cardiotoxic chemotherapy aims to ameliorate cardiovascular complications.^7^ As the prevalence of obesity, hypertension, and diabetes increases among children, adolescents, and young adults, consideration of CVRFs prior to diagnosis or during the early survivorship period is becoming more critical to personalized therapy aiming to reduce CVD risk.^8–12^ Indeed, CVRFs during childhood are associated with an increased risk of fatal and nonfatal cardiovascular events in adulthood.^13^

Previously identified disparities among adolescents and survivors from non-urban areas in survivorship care, such as optimal subspecialty follow-up and receipt of a survivorship care plan, may also influence downstream treatment-related CVD.^14^ This is particularly important for vulnerable populations, such as those from rural areas, are already at increased risk for CVRFS and CVD later in life.^15–17^ Moreover, fragmented healthcare systems for AYA survivors and those from non-urban areas underscore the role of data standards to surmount siloed data challenges through interoperability with the ultimate goal to improve patient care.^18^ Recent advances in data science, such as clinically computable phenotypes for hypertension and diabetes, offer strategies to leverage real world data and accelerate population health research.^19,20^

The objectives of this study were to implement a clinical informatics approach to identify CVRFs prior to or during cancer treatment among children, adolescents, and young adults (CAYA) with cancer and then analyze the impact of CVRFs on the subsequent development of CVD in the early survivorship period. As a secondary aim, survivors were linked to the Oklahoma Health Care Authority (OKHCA) data to evaluate potential inequities among survivors from non-urban areas and ameliorate underdetection bias from institutional data.

## METHODS

### Survivor Cohort Construction

The institutional cancer registry at the Stephenson Cancer Center at the University of Oklahoma reports all newly diagnosed cases to the National Cancer Database (NCDB), as per Commission on Cancer accreditation standards.^21,22^ The cancer registry contained the necessary demographic information (age at diagnosis, gender, and ZIP code to determine rurality). The cohort included children (ages 0–12 years), adolescents (ages 13–18 years), and young adults (ages 19–29 years) who, at the time of initial diagnosis, were evaluated in the academic pediatric oncology or medical oncology clinics, and received their first course of treatment at their respective centers. To reflect the reliability of cancer registry data for these age groups and ensure a longitudinal follow-up of five years, we included five-year survivors diagnosed between January 1, 2010, and December 31, 2017. This research was submitted to and approved by the University of Oklahoma Health Sciences Review Board (IRB#14731) on June 15, 2022.

### Disease Classification and Late Effects Risk Stratification

As part of NCDB standards, the International Classification of Diseases-Oncology, third edition (ICD-O3) was used to group diagnoses into primary malignancy categories based on the International Classification of Childhood Cancer, third edition (ICCC-3).^23^ Coding for bone tumors, central nervous system tumors, Hodgkin’s lymphoma, non-Hodgkins lymphoma, leukemia, neuroblastoma, retinoblastoma, sarcoma, Wilms tumor, and other categories were previously reported.^24^ The cancer registry captures whether patients received chemotherapy, surgery, radiation, or transplant as dichotomous variables.^21^ Late effects risk Stratification, based on primary diagnosis and dichotomous treatment exposures, was conducted based on the British Childhood Cancer Survivor Study risk groups.^25^

### Cardiovascular Risk Factors and Cardiovascular Disease

The Clinical Research Data Warehouse Team at the University of Oklahoma Health Sciences Center used standard query language to extract key data elements for CVRFs and race/ethnicity from the EHR. The primary CVRFs for this analysis included hypertension, diabetes, obesity, and hyperlipidemia. The Common Terminology Criteria for Adverse Events (CTCAE, v5.0) were used to classify CVRFs.^26^ For hypertension, CTCAE Grade ≥ 2 was defined as a diagnosis consistent with hypertension and an outpatient prescription for an antihypertensive medication (**Supplemental Material 1**). The Observational Medical Outcomes Partnership Common Data Model (OMOP CDM) provides a critical framework for reliable data standards and supports research with real-world data.^27,28^ For medication data, OMOP CDM utilizes RxNorm codes and previous research supports the utility of this model to classify antihypertensive medications automatically extracted from the EHR.^29^ We leveraged the RxNorm Concept Unique Identifier to ascertain survivors with outpatient prescriptions for antihypertensive medications prior to diagnosis or within the first year of initial diagnosis. Grade ≥ 2 diabetes was defined as an ICD-9/10 code consistent with diabetes or HgbA1C ≥ 6.5% from discrete observational lab data. For obesity, discrete data elements were extracted from the EHR to classify survivors as obese according to CTCAE Grade ≥ 3 with a body-mass index ≥ 30 (or > 95th percentile based on age-and sex-specific distributions) prior to diagnosis or within one year of initial diagnosis. Finally, dyslipidemia was defined based on ICD-9/10 coding. **Supplemental Material 1** provides a detailed account of the clinically computable phenotypes, based on discrete observations, ICD-9/10 codes, and RxNorm codes, used to define CVRFs for this analysis.

Heart failure or cardiomyopathy was the primary CVD outcome for this analysis. A previously published methodology, based on ICD-9/10 codes, was utilized (**Supplemental Material 2**).^13,30^ The date of initial diagnosis was used to landmark the date of diagnosis for CVD. Survivors with CVD prior to diagnosis or within one year of diagnosis were excluded from the analysis. An incident case of CVD was defined as an ICD-9/10 code consistent with cardiomyopathy or heart failure one to five years after the initial cancer diagnosis.

### Oklahoma Health Care Authority (OKHCA) Data

The cancer registry and institutional EHR data were linked to Medicaid records from OKHCA. Medicaid number was used as the primary Identifier for linkage and supplemented by other Identifiers such as date of birth, and first, and last names (for survivors without a match). The ICD-9/10 codes for CVD, as described above, were used and landmarked by date of cancer and CVD diagnosis to detect incident cases during the early survivorship period.

### Statistical analyses

Descriptive statistics including mean, standard deviation, median, and interquartile range (IQR) were calculated for continuous variables (age at diagnosis). Percentages and counts were calculated for categorical variables (age group, sex, race/ethnicity, rurality, primary diagnosis, late effects risk group, hypertension, diabetes, dyslipidemia, and obesity). The chi-square test was used to examine the association between each predictor and CVD status if all cell counts were greater than 5. Fisher’s exact test was used if any cell count was less or equal to 5. Unadjusted risk ratio (RR), RR adjusted for late effects risk strata and the corresponding 95% confidence intervals (CI) for examining the association between rurality and CVD in the early survivorship period were calculated using a modified Poisson regression model with robust error variance. Manual backward variable selection was used with an alpha threshold of 0.05. Confounding was assessed between predictors if the removal of one characteristic influenced a change of 20% or more in remaining characteristics. Collinearity was assessed with a threshold of 0.70.^31^ Missing values were excluded from the analysis, all of which were in the group without a cardiac event (0.3% were missing race/ethnicity, 0.7% were missing rurality, 3.3% were missing late effects risk Stratification due to incomplete exposure documentation). All analyses were performed by using SAS 9.4.

## RESULTS

### Cardiovascular Risk Factors and Cardiovascular Disease among Survivors

**Figure 1:**
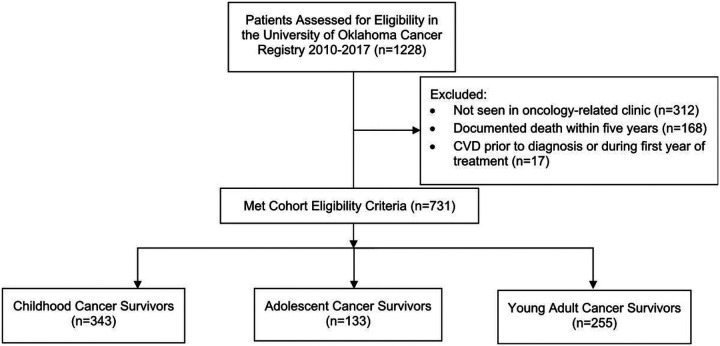
Childhood, Adolescent, and Young Adult Cancer Survivorship Cohort Construction

**Figure 2:**
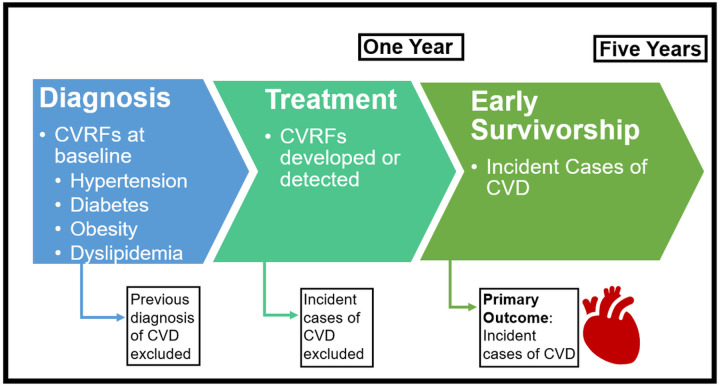
Temporal relationship between cardiovascular risk factors (CVRFs) at baseline or during treatment and the development of cardiovascular disease (CVD) in the early survivorship period

Between 2010 and 2017, there were 1228 children, adolescents, and young adults with cancer reported to the institutional cancer registry who completed their first course of treatment at the Jimmy Everest Center or the Stephenson Cancer Center. Among those with established oncology-related care (n = 916), an overall five-year survival of 82% was observed. The analytic cohort excluded those with early documented death (n = 168) and those not seen in an oncology-related clinic (n = 312) (Fig. 1). In order to establish a temporal relationship between CVRFs and the detection of CVD, survivors with CVD prior to or during the first year of treatment (n = 17) were also excluded (Fig. 2). Among the analytic survivor cohort, there were 10 incident cases (1.4%) of CVD observed between one year and five years after treatment. Grade ≥ 2 hypertension was observed in 106 survivors (14.5%), 37 met the criteria for diabetes (5.1%), eight survivors with dyslipidemia (1.1%), and 226 were obese (30.9%). All ten of the cardiac events were observed in survivors with OKHCA coverage while 67% of survivors without an event had OKHCA coverage (p = 0.04). Survivors at high, moderate, and low risk had a cumulative incidence of cardiac events of 5.2%, 1.1%, and 0.4%, respectively; the percent of patients in each risk category differed significantly between survivors with and without CVD (p = 0.01). There were no statistically significant associations between CVRFs and CVD in the early survivorship period (Table 1).

### Oklahoma Healthcare Authority Data Analysis

Data linkage of the analytic survivor cohort (n = 731) with OKHCA claims data showed that 67.7% of survivors had Medicaid coverage (n = 495). The inclusion of claims data identified two additional cases of CVD one to five years after initial diagnosis that were not captured by institutional data, which yielded a cumulative incidence of 2.4% (n = 12). Among survivors with OKHCA coverage, those from small town/isolated rural areas accounted for 50% of the incident cases of CVD, despite representing 17.5% of all survivors. Survivors from rural areas had a cumulative CVD incidence of 6.9% compared with 1.9% and 1.3% of survivors from large town and urban areas, respectively (p = 0.02). Similar to the full cohort, there was a significant association between late effects risk strata and CVD (p = 0.006). Demographics, such as age, gender, and race/ethnicity, as well as CVRFs were not significantly associated with CVD in the early survivorship period among those with OKHCA coverage (Table 2).

Individually significant predictors related to CVD were age, age group, rurality, and late effects risk strata. Age and age group at diagnosis had high collinearity (r = 0.83), thus continuous age was chosen as the preferred indicator for the model. However, age was not significantly related to CVD when included alongside other predictors and was dropped. Patient late effects risk strata was determined to be a confounder and is retained in the final model. Therefore, multivariable modified Poisson regression modeling showed that there was a persistent association between rurality and CVD, as survivors from small town/isolated rural areas had a 4.1 times greater risk (95% Confidence Interval 1.1–15.3) of CVD compared with survivors from urban areas after adjustment for late effects risk strata (Table 3).

A comparison of survivors with and without OKHCA coverage yielded several noteworthy differences (Table 4). Although primary diagnosis and late effects risk groups were not associated with OKHCA coverage, rurality was significantly associated with OKHCA coverage as 78.4% and 75.5% of survivors from small town/isolated rural areas and large towns had OKHCA coverage, respectively, compared with 63.7% of survivors from urban areas (p < 0.01). Moreover, race/ethnicity (p < 0.01), female sex (p = 0.03), and young adult age group at diagnosis (p = 0.02) significantly differed by OKHCA coverage status. Regarding CVRFs, 17% of survivors with coverage had hypertension compared with 9% of those without OKHCA coverage (p < 0.01). For obesity, prevalence was 33% and 26% among survivors with and without OKHCA coverage, respectively (p = 0.03).

## DISCUSSION

In this single institution cohort of CAYA survivors, clinical informatics tools based on discrete data elements from the EHR were leveraged to construct clinically computable phenotypes and evaluated the prevalence of CVRFs prior to diagnosis and during treatment. This represents a feasible approach to identify CVRFs on a population health level for at risk survivors. No significant associations were observed between CVRFs and CVD in the early survivorship period for this cohort, yet this analysis and methods inform efforts to harness real world data to drive improvement in survivorship-focused care. Furthermore, the presented analyses identified survivors at high risk for late effects in general and those with OKHCA coverage were at increased risk of CVD in the early survivorship period. Claims data augmented the detection of cardiac events among survivors with OKHCA coverage and the analysis from this subcohort suggested that those from rural areas were at increased risk of CVD even after adjustment for late effects risk strata. Rural-urban differences, particularly inequities in cardiovascular health, in the general population underscores the need to prevent CVD, particularly for CAYA survivors at risk for late morbidity and mortality.

The disproportionate burden of CVRFs and CVD in rural areas in the United States is well documented. In 2020, the American Heart Association released a call to action to reduce longstanding inequities in CVD among rural populations with a focus on individual factors, social determinants of health, and health delivery systems. Indeed, adults in rural areas demonstrate a higher risk of mortality from heart failure, at an individual and a community level, from population-based studies throughout the United States.^16,32–34^ The evidence of recent progress on closing the rural-urban gap is mixed, and the persistence of these geographic disparities in the general population should inform healthcare delivery interventions for survivors.^17,35^ Stratification by OKHCA coverage, particularly with the highlighted differences in race/ethnicity, rurality, hypertension, and obesity, further controls for these potential confounders and helps characterize this vulnerable population. The observed increased risk of CVD among survivors of CAYA cancer from rural areas in Oklahoma with public insurance suggests that, even as soon as one to five years after the initial diagnosis, there is an opportunity to intervene and mitigate risk.

Data science and the development of clinical informatics tools have the potential to catalyze improvements in health services research, guide population health management, and drive systems-level changes to promote equity for all survivors of CAYA. The presented methodology derived from data standards, such as RxNorm’s RxCUI codes for antihypertensive medications, and the novel creation of clinically computable phenotypes support the feasibility of such tools to characterize modifiable risk factors among survivors at a population health level.^29^ The analyses of the Oklahoma cohort failed to identify significant associations between CVRFs and CVD, which may reflect limitations in this cohort or perhaps suggest further refinement of clinically meaningful phenotypes to predict CVD are needed. Nevertheless, data standards are foundational to ensure the interoperability of key information between health systems, both from a research and clinical operations viewpoint.^36^ Moreover, the Childhood Cancer Data Initiative (CCDI) seeks to address the fragmented data ecosystem and has made progress toward an infrastructure to facilitate data sharing to learn from every child, adolescent, and young adult with cancer.^37^ More than a decade after the Health Information Technology for Economic and Clinical Health (HITECH) Act, lessons across the healthcare field in various specialties and domains offer insights to adapt evidence-based technologies for oncology and survivorship-focused care.^38,39^

The observations and analyses from the CAYA survivor cohort require contextualization for potential limitations. First, this cohort represented a single institution. While the majority of children in the state are treated at Oklahoma Children’s Hospital, young adults may have received treatment at community-based oncology centers and there is one other site in Oklahoma that cares for children with cancer. Therefore, the data may not be representative of the state of Oklahoma or generalizable to the national population. Data linkage with claims data uncovered rural-urban differences in CVD, which likely reflects detection bias from institutional data as the absence of diagnosis records does not necessarily mean the absence of disease.^40^ Alternatively, the observed differences may only exist in the Medicaid population. Underdetection of CVRFs, such as dyslipidemia or diabetes, is also possible if they are not routinely assessed or documented from EHR-based data. The lack of robust historical data prior to 2009 and moderate cohort size may have contributed to insufficient power to detect potential associations between CVRFs and CVD. Additionally, in this cohort, acute cardiotoxicity was observed and events within a year of diagnosis were excluded from analysis, as assessment of baseline CVRFs prior to diagnosis was likely incomplete and would have muddled the temporal relationship.

The long latency period for heart failure, specifically, poses a significant challenge to capture enough events to facilitate real world evidence for the association between baseline CVRFs and subsequent CVD in the early survivorship period.^41–43^ One approach to circumvent this long latency period is to identify early markers of cardiac dysfunction, such as echocardiogram parameters and cardiac biomarkers, which are useful predictors of subsequent CVD risk.^44–46^ Previously developed and validated NLP algorithms, such as EchoExtractor, serve as an example for open source informatics to automatically extract echocardiogram parameters.^47^ Left ventricular ejection fraction (LVEF) was the most commonly extracted echocardiogram measurement and the system has subsequently provided key data for population health studies on cardiac function, including the scalability of this system at multiple hospital sites.^48–50^ The sole reliance on ICD-9/10 coding, while based on methods from large multi-institutional cohorts, may also lead to misClassification of cardiac events, which could be amenable to more precise measurements from echocardiograms.^13^ Even with the implementation of such tools, underdetection bias may still persist if echocardiogram reports are unavailable. Adolescent survivors in Oklahoma were previously identified as approximately five times more likely to receive suboptimal guideline-adherent echocardiogram surveillance.^51^

In conclusion, clinical informatics tools to integrate data from various sources for cohort construction and apply data standards to characterize CVRFs highlight opportunities to leverage data to improve survivorship-focused care for CAYAs impacted by cancer. Survivors from rural areas may be at increased risk for CVD, even in the early survivorship period. Modifiable CVRFs at baseline and during treatment merit additional investigation to determine their impact on later CVD for survivors. This study provides a framework to adapt clinical informatics-based approaches for CAYA survivors to promote interoperability based on data standards, facilitate interinstitutional collaborations to detect relevant predispositions to CVD, and, ultimately, improve care for equitable outcomes among all survivors.

## Figures and Tables

**Table 1:** Cardiovascular Risk Factors During Treatment and Cardiovascular Disease in the Early Survivorship Period

	Cardiac Event		P-Value
	Yes (n = 10)	No (n = 721)	
Age, in years Mean (SD)	9.0 (8.0)	13.8 (9.5)	1.00
Median (IQR)	8.5 (13)	13 (18)	
Age Group at Diagnosis *Child*	5 (50%)	338 (47%)	0.08
*Adolescent*	4 (40%)	129 (18%)	
*Young Adult*	1 (10%)	254 (35%)	
Sex *Male*	6 (60%)	372 (52%)	0.22
*Female*	4 (40%)	349 (48%)	
Race/Ethnicity^a^ *White, Non-Hispanic*	7 (70%)	463 (64%)	0.33
*Black, Non-Hispanic; Hispanic; American Indian; Other*	3 (30%)	256 (26%)	
Rurality *Urban*	4 (40%)	472 (66%)	0.07
*Large Town*	2 (20%)	137 (19%)	
*Small Town/Isolated Rural*	4 (40%)	107 (15%)	
Oklahoma Health Care Authority Coverage *Yes*	10 (100%)	485 (67%)	0.04
*No*	0 (0%)	236 (33%)	
Primary Diagnosis^a^ *CNS*	3 (30%)	175 (24%)	0.31
*Leukemia and Lymphoma*	2 (20%)	240 (33%)	
*Neuroblastoma and Wilms Tumor*	3 (30%)	55 (8%)	
*Other*	2 (20%)	201 (28%)	
*Bone Tumor, Sarcoma, Retinoblastoma*	0 (0%)	50 (7%)	
Late Effects Risk Group^b^ *Low*	1 (10%)	244 (35%)	0.01
*Moderate*	4 (40%)	361 (52%)	
*High*	5 (50%)	92 (13%)	
Hypertension *Yes*	3 (30%)	103 (14%)	0.17
*No*	7 (70%)	618 (86%)	
Diabetes *Yes*	0 (0%)	37 (5%)	1.00
*No*	10 (100%)	684 (95%)	
Dyslipidemia *Yes*	0 (0%)	8 (1 %)	1.00
*No*	10 (100%)	713 (99%)	
Obesity *Yes*	2 (20%)	224 (31%)	0.73
*No*	8 (80%)	497 (69%)	

a Variables collapsed due to small numbers and concern for confidentiality

b Based on the British Childhood Cancer Survivor Study risk groups (Frobisher *et al* 2017)

**Table 2:** Cardiovascular Risk Factors During Treatment and Cardiovascular Disease in the Early Survivorship Period among Survivors with Oklahoma Healthcare Authority (OHCA) Coverage

	Cardiac Event		P-Value
	Yes (n = 12)	No (n = 483)	
Age, in years Mean (SD)	11.3 (9.1)	13.2 (9.4)	1.00
Median (IQR)	13 (17)	13 (18)	
Age Group at Diagnosis *Child*	5 (42%)	238 (49%)	0.43
*Adolescent*	4 (33%)	92 (19%)	
*Young Adult*	3 (25%)	153 (32%)	
Sex *Male*	6 (50%)	236 (49%)	0.47
*Female*	6 (50%)	247 (51 %)	
Race/Ethnicity^a^ *White, Non-Hispanic*	8 (67%)	289 (60%)	0.20
*Black, Non-Hispanic; Hispanic; American Indian; Other*	4 (33%)	192 (40%)	
Rurality *Urban*	4 (33%)	299 (62%)	0.02
*Large Town*	2 (17%)	103 (21 %)	
*Small Town/Isolated Rural*	6 (50%)	81 (17%)	
Primary Diagnosis^a^ *CNS*	4 (33%)	114 (24%)	0.32
*Leukemia and Lymphoma*	2 (17%)	174 (36%)	
*Neuroblastoma and Wilms Tumor*	3 (25%)	36 (7%)	
*Other*	3 (25%)	132 (27%)	
*Bone Tumor, Sarcoma, Retinoblastoma*	0 (0%)	27 (6%)	
Late Effects Risk Group *Low*	2 (17%)	171 (37%)	0.006
*Moderate*	4 (33%)	236 (51 %)	
*High*	6 (50%)	59 (12%)	
Hypertension *Yes*	3 (25%)	81 (17%)	0.44
*No*	9 (75%)	402 (83%)	
Diabetes *Yes*	0 (0%)	27 (6%)	1.00
*No*	12 (100%)	456 (94%)	
Dyslipidemia *Yes*	0 (0%)	5 (1 %)	1.00
*No*	12 (100%)	478 (99%)	
Obesity *Yes*	3 (25%)	161 (33%)	0.76
*No*	9 (75%)	322 (67%)	

a Variables collapsed due to small numbers and concern for confidentiality

b Based on the British Childhood Cancer Survivor Study risk groups (Frobisher *et al* 2017)

**Table 3:** Modeling for Association between Rurality and CVD in the Early Survivorship Period

RUCA	Unadjusted Risk Ratio (95% Confidence Interval)	Adjusted^1^ Risk Ratio (95% Confidence Interval)
Large Town vs. Urban	1.71 (0.32–9.26)	1.46 (0.27–7.84)
Small Town/Isolated Rural vs. Urban	4.45 (1.13–17.51)	4.09 (1.10–15.28)

1 Adjusted for late effects risk strata

**Table 4:** Comparison of Survivors with and without OKHCA Coverage

	Yes (n = 495)	No (n = 236)	p-value
Age, in years Mean (SD)	13.16 (9.38)	14.88 (9.57)	0.19
Median (IQR)	13 (4–22)	15 (5–24)	
Age Group at Diagnosis *Child*	243 (70.85%)	100 (29.15%)	0.02
*Adolescent*	156 (61.18%)	99 (38.82%)	
*Young Adult*	96 (72.17%)	37 (27.82%)	
Sex *Male*	242 (64.02%)	136 (35.98%)	0.03
*Female*	253 (71.67%)	100 (28.33%)	
Race/Ethnicity *White, Non-Hispanic*	297 (63.19%)	173 (36.81%)	<0.01
*Black, Non-Hispanic*	49 (83.05%)	10 (16.95%)	
*Hispanic*	87 (74.36%)	30 (25.64%)	
*American Indian*	44 (78.57%)	12 (21.43%)	
*Other*	16 (59.26%)	11 (40.74%)	
Rurality *Urban*	303 (63.66%)	173 (36.34%)	<0.01
*Large Town*	105 (75.54%)	34 (24.46%)	
*Small Town/Isolated Rural*	87 (78.38%)	24 (21.63%)	
Primary Diagnosis *Bone Tumor*	14 (58.33%)	10 (41.67%)	0.33
*CNS*	118 (66.29%)	60 (33.71%)	
*Hodgkins Lymphoma*	23 (57.50%)	17 (42.50%)	
*Non-Hodgkins Lymphoma*	24 (66.67%)	12 (33.33%)	
*Leukemia*	129 (77.71%)	37 (22.29%)	
*Neuroblastoma*	22 (75.86%)	7 (24.14%)	
*Retinoblastoma*	5 (83.33%)	1 (16.67%)	
*Sarcoma*	8 (40%)	12 (60%)	
*Wilms Tumor*	17 (2.33%)	12 (1.64%)	
*Other*	135 (66.50%)	68 (33.50%)	
Late Effects Risk Group *Low*	173 (70.61%)	72 (29.39%)	0.45
*Moderate*	240 (65.75%)	125 (34.25%)	
*High*	65 (67.01%)	32 (32.99%)	
Hypertension *Yes*	84 (79.25%)	22 (20.75%)	<0.01
*No*	411 (65.76%)	214 (34.24%)	
Diabetes *Yes*	27 (72.97%)	10 (27.03%)	0.48
*No*	468 (67.44%)	226 (32.56%)	
Dyslipidemia *Yes*	5 (62.50%)	3 (37.50%)	0.27
*No*	490 (67.77%)	233 (32.23%)	
Obesity *Yes*	164 (72.57%)	62 (27.43%)	0.03
*No*	331 (65.54%)	174 (34.46%)	

## Data Availability

The data that support the findings of this study are available on request from the corresponding author. The data are not publicly available due to privacy or ethical restrictions.
